# A Critical Role for Astrocytes in Hypercapnic Vasodilation in Brain

**DOI:** 10.1523/JNEUROSCI.0005-16.2016

**Published:** 2017-03-01

**Authors:** Clare Howarth, Brad Sutherland, Hyun B. Choi, Chris Martin, Barbara Lykke Lind, Lila Khennouf, Jeffrey M. LeDue, Janelle M.P. Pakan, Rebecca W.Y. Ko, Graham Ellis-Davies, Martin Lauritzen, Nicola R. Sibson, Alastair M. Buchan, Brian A. MacVicar

**Affiliations:** ^1^Djavad Mowafaghian Centre for Brain Health, University of British Columbia, Vancouver, British Columbia V6T 1Z3, Canada,; ^2^Cancer Research United Kingdom and Medical Research Council Oxford Institute for Radiation Oncology, Department of Oncology, University of Oxford, Oxford, OX3 7DQ, United Kingdom,; ^3^Acute Stroke Programme, Radcliffe Department of Medicine, University of Oxford, Oxford, OX3 9DU, United Kingdom,; ^4^Department of Psychology, University of Sheffield, Sheffield, S10 2TP, United Kingdom,; ^5^Department of Neuroscience and Pharmacology and Center for Healthy Aging, University of Copenhagen, DK-2200 Copenhagen N, Denmark,; ^6^Department of Neuroscience, Mount Sinai School of Medicine, New York, New York 10028, and; ^7^Department of Clinical Neurophysiology, Rigshospitalet, DK-2600 Glostrup, Denmark

**Keywords:** astrocyte, calcium, cerebral blood flow, glutathione, hypercapnia

## Abstract

Cerebral blood flow (CBF) is controlled by arterial blood pressure, arterial CO_2_, arterial O_2_, and brain activity and is largely constant in the awake state. Although small changes in arterial CO_2_ are particularly potent to change CBF (1 mmHg variation in arterial CO_2_ changes CBF by 3%–4%), the coupling mechanism is incompletely understood. We tested the hypothesis that astrocytic prostaglandin E_2_ (PgE_2_) plays a key role for cerebrovascular CO_2_ reactivity, and that preserved synthesis of glutathione is essential for the full development of this response. We combined two-photon imaging microscopy in brain slices with *in vivo* work in rats and C57BL/6J mice to examine the hemodynamic responses to CO_2_ and somatosensory stimulation before and after inhibition of astrocytic glutathione and PgE_2_ synthesis. We demonstrate that hypercapnia (increased CO_2_) evokes an increase in astrocyte [Ca^2+^]_i_ and stimulates COX-1 activity. The enzyme downstream of COX-1 that synthesizes PgE_2_ (microsomal prostaglandin E synthase-1) depends critically for its vasodilator activity on the level of glutathione in the brain. We show that, when glutathione levels are reduced, astrocyte calcium-evoked release of PgE_2_ is decreased and vasodilation triggered by increased astrocyte [Ca^2+^]_i_ *in vitro* and by hypercapnia *in vivo* is inhibited. Astrocyte synthetic pathways, dependent on glutathione, are involved in cerebrovascular reactivity to CO_2_. Reductions in glutathione levels in aging, stroke, or schizophrenia could lead to dysfunctional regulation of CBF and subsequent neuronal damage.

**SIGNIFICANCE STATEMENT** Neuronal activity leads to the generation of CO_2_, which has previously been shown to evoke cerebral blood flow (CBF) increases via the release of the vasodilator PgE_2_. We demonstrate that hypercapnia (increased CO_2_) evokes increases in astrocyte calcium signaling, which in turn stimulates COX-1 activity and generates downstream PgE_2_ production. We demonstrate that astrocyte calcium-evoked production of the vasodilator PgE_2_ is critically dependent on brain levels of the antioxidant glutathione. These data suggest a novel role for astrocytes in the regulation of CO_2_-evoked CBF responses. Furthermore, these results suggest that depleted glutathione levels, which occur in aging and stroke, will give rise to dysfunctional CBF regulation and may result in subsequent neuronal damage.

## Introduction

Astrocyte [Ca^2+^]_i_ transients have been shown to directly alter diameters of cerebral arterioles in experiments using either direct astrocyte stimulation or calcium uncaging in astrocytes of juvenile (Zonta et al., 2003; Mulligan and MacVicar, 2004; Gordon et al., 2008), or adult animals (Takano et al., 2006). However, several laboratories have published contradictory evidence on whether, in adult animals, astrocyte [Ca^2+^]_i_ signaling is evoked by synaptic activity leading to neurovascular coupling (Zonta et al., 2003; Petzold et al., 2008; Schulz et al., 2012; Lind et al., 2013; Otsu et al., 2015) or not (Nizar et al., 2013; Takata et al., 2013; Bonder and McCarthy, 2014). More recently, astrocyte [Ca^2+^]_i_ was shown to modify basal arteriole tone in adult animals (Rosenegger et al., 2015). Therefore, it is still poorly understood when, how, and under what conditions, astrocyte [Ca^2+^]_i_ signaling contributes to the regulation of cerebral blood flow (CBF).

In this work, we investigated the mechanisms underlying CBF responses to increased blood CO_2_ concentrations (hypercapnia) and the potential contribution of astrocytes to those CBF responses. Arterial CO_2_ has a potent effect on CBF, with a 1 mmHg variation eliciting a 3%–4% CBF change (Hauge et al., 1980). However, the mechanism coupling a change in CO_2_ to a change in CBF is incompletely understood. There are parallels between the vasoactive signals generated by astrocytes and those underlying hypercapnia-evoked CBF responses. Astrocytes have been shown to directly modify arteriole diameter when their intracellular [Ca^2+^]_i_ increases, activating astrocytic phospholipase A_2_ (PLA_2_) (He et al., 2012) and thereby generating arachidonic acid (AA) and several vasoactive metabolites including PgE_2_, which causes vasodilation (Zonta et al., 2003; Takano et al., 2006; Gordon et al., 2008; Attwell et al., 2010). In addition to their roles in neurovascular coupling, both PgE_2_ (Wagerle and Mishra, 1988; Wagerle and Degiulio, 1994) and cyclooxygenase-1 (COX-1) activity (Niwa et al., 2001) are involved in increasing CBF during hypercapnia. We examined the potential link between astrocytes and increased CBF during hypercapnia because astrocytes express the enzymes that are involved in synthesizing PgE_2_ from AA during hypercapnia-induced CBF changes (Niwa et al., 2001). For example, mRNA for both COX-1 and microsomal prostaglandin E synthase-1 (mPgES-1) are reported in transcriptome studies to be highly expressed in astrocytes but not neurons (e.g., ptgs1, also known as COX-1, is 15-fold higher in astrocytes than in neurons) (Cahoy et al., 2008; Zhang et al., 2014). Astrocytes are immunoreactive for both the enzyme proteins COX-1 (Takano et al., 2006; Gordon et al., 2008) and mPgES-1 (see Fig. 3*A* and Tachikawa et al., 2012). mPgES-1, the form of prostaglandin E synthase expressed in astrocytes, requires the cofactor glutathione (GSH) (Jakobsson et al., 1999; Murakami et al., 2000) that is present in high levels in astrocytes (see Fig. 3*B* and Sun et al., 2006; Bragin et al., 2010; Robillard et al., 2011). We investigated whether hypercapnia can evoke an increase in astrocyte [Ca^2+^]_i_ *in vivo* and, if so, whether this results in activation of a PgE_2_-mediated vasodilation. In doing so, we demonstrate a novel, GSH-dependent mechanism of CBF regulation, which involves astrocytes and the GSH-sensitive release of PgE_2_.

## Materials and Methods

### 

#### Slice preparation

Four hundred μm hippocampal-neocortical slices were prepared from male and female juvenile (postnatal age 16–21 d) Sprague Dawley rats. Treatment of animals was approved by the University of British Columbia Animal Care and Use Committee. As previously described (Gordon et al., 2008), rats were anesthetized with halothane, decapitated, and the brains removed into ice-cold slicing solution containing the following (in mm): 2.5 KCl, 26 NaHCO_3_, 0.5 CaCl_2_, 10 MgSO_4_, 1.25 NaH_2_PO_4_, 10 glucose, 230 sucrose, saturated with 95% O_2_/5% CO_2_. The 400 μm transverse hemi-sections were incubated at 32°C-34°C in aCSF containing the following (in mm): 126 NaCl, 2.5 KCl, 26 NaHCO_3_, 2.0 CaCl_2_, 2.0 MgCl_2_, 1.25 NaH_2_PO_4_, 10 glucose, saturated with 95% O_2_/5% CO_2_ for 60 min. For experiments, slices were at 22°C-24°C, aCSF was saturated with 20% O_2_/5% CO_2_, balanced N_2_, and perfused at ∼2 ml/min. Healthy slices can be maintained in 20% O_2_, which provides a pO_2_ at the low end of the physiological range (Gordon et al., 2008). Astrocytes were loaded with the caged IP_3_ compound, NV-IP_3_/AM (5 μg/ml), and/or the Ca^2+^ indicator rhod-2/AM (10 μm, Invitrogen) as previously described (Mulligan and MacVicar, 2004; Gordon et al., 2008). Slices were loaded with monochlorobimane (MCB, Fluka) in the dark at room temperature for 30 min as previously described (Robillard et al., 2011).

#### Two-photon imaging and uncaging in acute brain slices

A two-photon laser-scanning microscope (Zeiss LSM510-Axioskop-2 fitted with a 40×-W/1.0 numerical aperture objective lens) coupled to a Chameleon ultra II Ti:sapphire laser (∼140 fs pulses 80 MHz, Coherent) provided excitation of rhod-2 and was used to uncage IP_3_. Images were acquired 50–100 μm below the slice surface. Rhod-2 fluorescence imaging and two-photon uncaging were performed using laser settings and emission filters as previously described (Gordon et al., 2008). MCB was excited at 780 nm and detected with a PMT at 512–562 nm as previously described (Robillard et al., 2011). Arterioles (defined as vessels with diameter >10 μm, surrounded by a visible layer of smooth muscle cells) were imaged by acquiring the transmitted laser light and using IR-DIC optics.

#### Glutathione and PgE_2_ measurements

Protocols in suppliers' instructions were followed for the PgE_2_ ELISA and glutathione assays. When measuring PgE_2_ release from acute brain slices, TTX (1 μm, Alamone Labs) was added to dampen neuronal activation. PgE_2_ release from acute brain slices was measured using a Specific Parameter PgE_2_ ELISA kit (R&D systems). Measurements of tissue glutathione levels were made using a specific total glutathione assay kit from either BioVision or Assay Designs.

#### Immunohistochemistry

Rats were anesthetized with halothane, given an intraperitoneal injection of urethane (0.5 ml of 30% urethane per 50 g body weight), and perfused with saline (0.9% NaCl in 0.1 m phosphate buffer) followed by 4% PFA (in 0.1 m PBS). The brain was extracted, postfixed (10% sucrose in 4% PFA) overnight, and cryoprotected (30% sucrose in PBS) overnight. Using a cryostat, 40 μm serial sections in the horizontal plane were collected throughout the brain. Free-floating sections were blocked with 10% normal goat serum (Jackson ImmunoResearch Laboratories) and 0.4% Triton X-100 in PBS for 1 h and incubated in PBS containing 0.1% Triton X-100 and primary antibodies against PgE_2_ synthase (anti-mPgES-1) (Olajide et al., 2014; Tuure et al., 2015) (Agrisera, catalog #AS03 031, 1:200) as well as an astrocyte phenotypic marker (anti-GFAP (Lathia et al., 2008) (Sigma, catalog #G3893, clone #G-A-5, 1:500) overnight at 4°C. Tissue was rinsed and incubated in AlexaFluor-488 goat anti-mouse and AlexaFluor-546 goat anti-rabbit secondary antibodies (Invitrogen: diluted 1:500 in PBS, 2.5% normal goat serum and 0.4% Triton X-100) for 1.5 h at room temperature. The tissue was rinsed, mounted onto slides, and coverslipped using Fluorsave mounting medium (Calbiochem). Images were acquired with an Olympus Fluoview FV1000 confocal microscope.

#### Drugs

trans-ACPD (tACPD), clonidine, norepinephrine (NE; Sigma), and PgE_2_ (Cayman Chemicals) were bath applied for 5–10 min. SC560 (Sigma) was preincubated for 30 min (Blanco et al., 2008) followed by bath application and buthionine sulfoximine (BSO; Sigma) was preincubated for 2.5 h (Sun et al., 2006) followed by bath application throughout the experiment. NV-IP_3_/AM was synthesized by G. Ellis-Davies.

#### Animals: *in vivo* blood flow measurements in rats

All procedures were approved by the University of Oxford Ethical Review Committee and complied with the requirements of the Animals (Scientific Procedures) Act, 1986, United Kingdom. Animals were housed in an animal housing facility in a 12 h alternating light:dark cycle with *ad libitum* access to food and water. Male Wistar rats were used (256–367 g).

#### Intracerebral injection

For surgical procedures, rats were anesthetized with 4% isoflurane and maintained at 1.5%–2% isoflurane in 30% O_2_ and 70% N_2_. Each rat was placed in a stereotaxic frame and the skull exposed. A burr hole was drilled at 1 mm caudal and 4.2 mm lateral to bregma, and the dura mater was finely dissected away to expose the cortex. Twenty μl of 80 mg/ml BSO (Pileblad and Magnusson, 1989) or 0.9% saline was infused by a microinfusion pump at a rate of 2 μl/min into the right whisker barrel cortex at a depth of 2.3 mm from the brain's surface. This dose of BSO has previously been shown to adequately reduce GSH within 24 h of administration (Pileblad and Magnusson, 1989), and we showed that BSO administered in this way could decrease GSH levels in the ipsilateral cortex by 45%, 24 h after injection (see Fig. 5*C*), and in the ipsilateral striatum by 31% (GSH measured in saline-treated: 0.61 ± 0.03 mM, BSO-treated: 0.42 ± 0.08 mM, *p* = 0.045, *n* = 7 per group, mean ± SEM). After the infusion, bone wax was placed over the burr hole and the wound was closed with 3–0 sutures. Animals recovered for 24 h before assessment of GSH levels (*n* = 7 per group) or evoked blood flow responses (*n* = 6–10 per group).

#### Whisker pad stimulation and hypercapnia: *in vivo* blood flow measurements

At 24 h after BSO/saline treatment, animals had their left femoral artery cannulated for blood gas measurement and were tracheotomized and ventilated with 1.25% isoflurane in 30% O_2_ and 70% N_2_. A laser Doppler probe (Perimed) to monitor relative CBF was placed over the right whisker barrel cortex (where the intracerebral injection was made) and bipolar stimulating electrodes were placed in the left whisker pad. For some experiments, a local field potential (LFP) electrode for neuronal activity was also placed on the exposed cortex to monitor neuronal activity. All animals had a steady-state blood gas (Table 1) before beginning experiments.

**Table 1.** Blood gases for BSO experiment (Nota Bene blood gases taken 24 h after drug but before hypercapnia and whisker stimulation experiments)*^a^*

Treatment	pH	pCO_2_ (mmHg)	pO_2_ (mmHg)
Saline	7.47 (0.01)	34.5 (2.3)	161 (4)
BSO*^b^*	7.46 (0.01)	35.8 (1.5)	154 (7)

*^a^*Data are mean (SEM).

*^b^*An inhibitor of γ-glutamylcysteine synthetase.

An electrical stimulus (10 Hz, 16 s duration, 1.6 mA, 0.3 ms pulsewidth, 60 s interstimulation interval) to evoke a blood flow response in the right whisker barrel cortex was performed for 10 trials per animal. Following this, animals were exposed to 10% CO_2_ for 30 s at 3 min intervals repeated 4 or 5 times to induce hypercapnic blood flow responses. Animals were killed and the cortex dissected for measurement of GSH levels.

For SC560 experiments, naive rats were anesthetized with isoflurane. Anesthesia was induced with 4% isoflurane and maintained during surgery with 2% isoflurane. During stimulation, anesthesia was maintained with 1.25% isoflurane. Isoflurane was carried in 30% O_2_ and 70% N_2_. Rats had their left femoral artery and vein cannulated, and were also tracheotomized and ventilated. A laser speckle camera (Moor Instruments) was used to monitor relative CBF over a thin skull window over the right whisker barrel cortex while an LFP electrode for neuronal activity was inserted through a burr hole. Bipolar stimulating electrodes were placed in the left whisker pad. Animals had a steady-state blood gas before and after drug administration (Table 2). An electrical stimulus (10 Hz, 16 s duration, 1.6 mA, 0.3 ms pulsewidth, 60 s interstimulation interval) to evoke a blood flow response in the right whisker barrel cortex was performed for 10 trials per animal. Following this, animals were exposed to 10% CO_2_ for 30 s at 3 min intervals repeated four times to induce a hypercapnic blood flow response. Animals were then administered 5 mg/kg SC560 or 10% DMSO (vehicle) intravenously. SC560 is a highly lipophilic COX-1 inhibitor and distributes widely into tissues (Teng et al., 2003), and this dose was chosen for maximal target efficiency (Zhang et al., 2003). After 20 min, the effect of COX-1 inhibition on the evoked CBF responses to whisker stimulation and hypercapnia was measured.

**Table 2.** Blood gases for SC560 intravenous experiment (Nota Bene blood gases taken before and after drug administration)*^a^*

Condition	Treatment	pH	pCO_2_ (mmHg)	pO_2_ (mmHg)
Predrug	DMSO	7.47 (0.01)	33.5 (1.5)	164 (5)
	SC560	7.45 (0.01)	36.8 (1.2)	140 (5)
Postdrug	DMSO	7.46 (0.01)	34.1 (0.6)	156 (6)
	SC560	7.45 (0.03)	37.1 (2.1)	140 (5)

*^a^*Data are mean (SEM).

#### Animals: *in vivo* calcium imaging

For *in vivo* experiments, all procedures involving animals were approved by the Danish National Ethics Committee according to the guidelines set forth in the European Council's Convention for the Protection of Vertebrate Animals used for Experimental and Other Scientific Purposes. The 8- to 10-week-old male C57BL/6J mice were used.

#### *In vivo* calcium imaging

For experiments involving mice, anesthesia was induced with bolus injections of the α2-adrenergic receptor agonist xylazine (10 mg/kg i.p.) and the NMDA-receptor antagonist ketamine (60 mg/kg i.p.). Anesthesia was maintained during surgery with supplemental doses of ketamine (30 mg/kg/20 min i.p.). Upon completion of all surgical procedures, anesthesia was switched to continuous infusion with α-chloralose (50 mg/kg/h i.v.).

Calcium activity during hypercapnia was measured *in vivo* in eight C57BL/6J mice. A craniotomy over the somatosensory cortex was covered with agarose and partly sealed with a glass coverslip. Oregon Green Bapta-1/AM (OGB; Invitrogen) was dissolved in DMSO and Pluronic F-127 (10%, BASF Global) and diluted in aCSF to yield a final dye concentration of 0.8 mm. It was mixed with the astrocyte marker sulforhodamine 101 (SR101; Sigma-Aldrich, 100 μm) (Nimmerjahn et al., 2004) and was pressure injected (4–6 psi, 4 s) into the somatosensory cortex through a micropipette at a depth of 100–150 μm below the cortical surface. Ca^2+^ imaging was performed using a commercial two-photon microscope (SP5 multiphoton/confocal Laser Scanning Microscope; Leica), and a Mai Tai HP Ti:sapphire laser (Millennia Pro, Spectra Physics) with a 20× 1.0 NA-water-immersion objective (Leica). The excitation wavelength was 820 nm. The emitted light was filtered to retain both red and green light using a TRITC/FITC filter.

The hypercapnia challenge was presented as follows: Following 1 min baseline recording, 10% CO_2_ in air was applied for 30 s and imaging continued for subsequent 4 min. Five trials were performed with 3 min between trials. For each animal, a second field of view was selected and the hypercapnia challenge repeated. Blood gases were taken after each experiment, and all mice had pCO_2_ in the range 30–40 mmHg and pO_2_ in the range 95–130 mmHg.

#### Data collection, analysis, and statistics

##### *In vitro* data.

An image (512 × 512 pixels) was collected in 7.86–12.68 s, using 8-line averaging. Measurements of lumen diameter and Ca^2+^ changes were performed offline with Zeiss LSM (version 3.2) software and ImageJ (National Institutes of Health). As previously described (Gordon et al., 2008), fluorescence signals were defined as *F*/*F*_0_ (%) = [(*F*_1_ − *B*_1_)/(*F*_0_ − *B*_0_)]100, where *F*_1_ and *F*_0_ are fluorescence at a given time and the mean fluorescence during the control period, respectively. *B*_1_ and *B*_0_ are the corresponding background fluorescence signals, taken from the neuropil. Pseudo-color images show absolute changes in fluorescence (ImageJ, 16-color linear Lut). Experimental values are mean ± SEM; *n* is the number of experiments conducted or, for calcium changes, number of astrocytes analyzed. Either a two-tailed Student's *t* test or a one-way ANOVA with a Newman–Keuls *post hoc* test for comparison between multiple groups was used, and *p* < 0.05 was considered statistically significant. As these were novel experiments, the effect size was unknown before the experiment. Therefore, sample size estimates were based on our previous experience. Experiments were alternately performed under control or treatment conditions with slices chosen at random for each experiment. Data were excluded from analysis if any of the following occurred during imaging: unstable baseline vessel diameters or astrocyte calcium levels, or movement leading to significant focus changes during the experiment. To perform statistical analysis, data were assumed to be normally distributed.

##### *In vivo* data.

All laser Doppler and LFP data were collected in Spike 2 software, whereas laser speckle data were collected using Moor FLPI software. Quantification of CBF changes and electrophysiology were performed in MATLAB (The MathWorks, version 7.12). To obtain the region of interest (ROI) for calculation of CBF changes using laser speckle imaging, a principal components analysis was used to identify the focal point of the change in response to stimulation. The same region of interest was used within each animal's data. Experimental values are the mean ± SEM, and *n* is the number of animals. To perform statistical analysis, data were assumed to be normally distributed. An *F* test was used to compare variances of groups being statistically compared. For CBF data, a one-tailed *t* test with Welch's correction (as groups had significantly different variances) was used to compare means between groups. A two-tailed *t* test was used to compare means of groups for both GSH analysis (see Fig. 5*C*) and electrophysiology data in response to whisker pad stimulation (Welch-corrected for SC560 experiment, see Fig. 6*C*). For electrophysiology data collected during hypercapnia challenge experiments, a two-way ANOVA with Bonferroni correction for multiple comparisons was used to compare means between groups. *p* < 0.05 was considered statistically significant. For experiments involving rats, due to effect sizes being unknown before experiment, sample size estimates were based on previously published sample sizes (e.g., Niwa et al., 2001). Assignment of animals was alternated between treatment and control groups, and neither experiments nor analysis were blinded. Three animals were excluded from all data analysis (1 for SC560 and 2 for BSO) due to technical problems with experimental equipment.

For *in vivo* calcium imaging, frame size was 256 × 256 pixels (189–207 ms/frame) during recordings. The Ca^2+^ changes were evaluated as the average change in fluorescence relative to baseline levels in ROIs. The ROIs were placed based on morphology over neuronal or astrocytic soma, or neuropil. Because of movement of astrocytes during hypercapnia, within or out of focus, ROIs were evaluated based on the level of SR101 loading in the red channel. If a significant change occurred, the ROI was disregarded in all following assessments. An increase in fluorescence within an ROI was classified as a calcium response if the mean fluorescence value within the period of hypercapnia was >2 SDs of baseline activity. The delay of the Ca^2+^ response was found by subtracting the signal onset time from the time hypercapnia was introduced to the animal. To estimate response start and termination time, a fit was made to the data and the first- and second-order derivatives were calculated. The response onset time was found by taking the maximum peak of the second order derivative of the fitted data. The duration of the Ca^2+^ response was then found by subtracting the response onset time from the response termination time. The response termination time was defined as the time point when the fitted data went below mean baseline Ca^2+^ levels or the recording ended. Experimental values are expressed as mean ± SEM. A paired *t* test was used for the calcium imaging data, each animal served as its own control. *p* < 0.05 was accepted as statistically significant. For experiments involving mice, as there have been no previous studies reporting astroglial calcium changes during hypercapnia, it was impossible to estimate an expected value for change in fluorescence or its SD. Hence, no sample size calculation could be performed. However, we expected similar calcium changes to those we observe for low-frequency whisker stimulation, and so sample sizes were based on our previous experiments (6–8 mice). Calcium signals obtained during hypercapnia exceeded an SNR of 4:1, and hypercapnia-induced calcium responses were recorded in every animal tested. As all mice were subjected to hypercapnia, there was no randomization method used. Control measurements of calcium activity (i.e., activity without application of hypercapnia) were taken at random time points during the experiment. Analysis of calcium changes was not blinded, assessment of these changes was based on a MATLAB program, which analyzes the image sequences in an unbiased manner, rather than by visual inspection.

## Results

### Increased CO_2_ evokes [Ca^2+^]_i_ responses in astrocytes *in vivo*

Elevation of tissue CO_2_ concentration, which can be caused by neuronal metabolism, is known to dilate cerebral blood vessels in a process dependent on PgE_2_ (Wagerle and Mishra, 1988; Wagerle and Degiulio, 1994) formation via COX-1 activity (Niwa et al., 2001). However, the cells that both are responsible for sensing CO_2_ and that also express the enzymes for synthesizing PgE_2_ (COX-1 and PgES) have not been resolved. Astrocytes can produce PgE_2_, but it is unknown whether astrocytes generate [Ca^2+^]_i_ signals in response to CO_2_. Therefore, we tested whether an increase in inspired CO_2_ (hypercapnia) *in vivo* evokes astrocyte [Ca^2+^]_i_ when it also triggers CBF increases.

**Figure 1.**
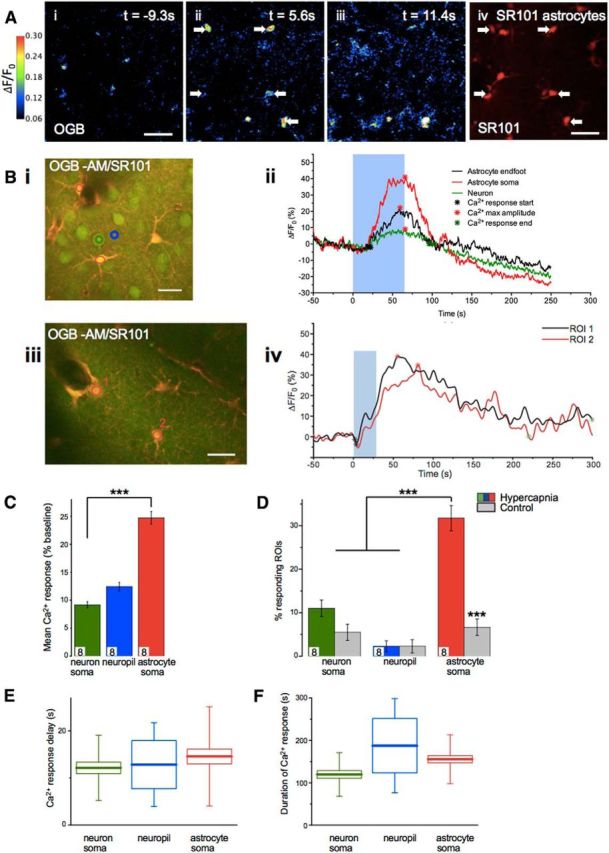
Astrocyte [Ca^2+^]_i_ transients are evoked by CO_2_ *in vivo*. ***A***, Example still images of mouse cortical layer II/III from 2PLSM. OGB is used as a calcium indicator (***Ai–Aiii***) and sulforhodamine 101 (SR101, ***Aiv***, average image for whole recording) is used to stain astrocytes. Color scale refers to images ***Ai–Aiii***. White arrows indicate astrocytes that show a Ca^2+^ response to CO_2_ of at least twice its baseline Ca^2+^ fluctuation. In this case, CO_2_ stimulus begins at *t* = 0 s and is applied for 36 s. ***Aiii***, Recovery of immediate CO_2_ induced Ca^2+^ transient. Scale bars, 40 μm. ***Bi***, ***Biii***, Further example images of mouse cortical layer II/III from 2PLSM showing example ROI placement. Merge images showing OGB and SR101 (***Bi***, ***Biii***). Red ROI1 indicates astrocyte endfoot. Red RO12 indicates astrocyte soma (layer II: *n* = 181, 8 mice). Green ROI indicates neuron soma (layer II: *n* = 153, 8 mice). Blue ROI indicates neuropil (layer II: *n* = 104, 8 mice). Scale bar, 20 μm. Example time series (***Bii***, ***Biv***) of [Ca^2+^]_i_ response in astrocyte and neuron soma ROIs (as indicated in ***Bi***, ***Biii***). Blue box represents time during which expired CO_2_ level is increased. ***C***, Mean Ca^2+^ response in ROIs. Colors represent ROIs located as shown in ***Bi***. ***D***, Percentage of ROIs for each cell type that showed a Ca^2+^ response with and without a hypercapnia stimulus. For no hypercapnia (control), *n* = 170 astrocyte somas, *n* = 148 neuronal soma, and *n* = 96 neuropil ROIs, *n* = 8 mice. Colors represent description in ***B***. ***E***, Delay from hypercapnia start time to start of Ca^2+^ response in ROI. ***F***, Duration of Ca^2+^ response in each ROI in response to CO_2_ stimulus. ***E***, ***F***, Box plots represent the mean (small square). Edges of the box represent 25% and 75% of data. End lines indicate maximum and minimum values. Data are mean ± SEM. **p < 0.01. ***p < 0.001.

Two-photon laser scanning microscopy (2PLSM) *in vivo* was used to examine the simultaneous responses of both neurons and astrocytes to hypercapnia in the intact brain as a first step to investigate which cell type might be the primary sensor of CO_2_ (Fig. 1). Remarkably, we found consistent and significant increases in [Ca^2+^]_i_ in the soma and endfeet of astrocytes in cortical layers II/III of mouse (Fig. 1) during the period of hypercapnia. The dramatic increases that we observed in astrocytes were of significantly higher amplitude (Fig. 1*A–C*; *p* < 0.01) than increases in [Ca^2+^]_i_ observed in neuronal soma during the period of hypercapnia. The number of astrocytes with [Ca^2+^]_i_ responses was also much greater in hypercapnia compared with the number showing spontaneous calcium activity (control time period: Fig. 1*D*; *p* < 0.01). Although neurons could display increased [Ca^2+^]_i_ during hypercapnia, with onset times within seconds (Fig. 1*B*,*C*,*E*), there was no significant difference in the number of neurons with [Ca^2+^]_i_ responses during hypercapnia compared with the number showing spontaneous calcium activity (control time period: Fig. 1*D*). Measurements taken in the neuropil where there were no defined cell bodies, and it is difficult to separate signals in fine astrocyte processes from neuronal processes did not show correlated changes in [Ca^2+^]_i_ signals during hypercapnia (Fig. 1*D*). The astrocyte [Ca^2+^]_i_ responses (Fig. 1*B,E*,*F*) appear to occur within a similar timescale as the increased CBF evoked by hypercapnia (as measured by laser speckle contrast imaging and laser Doppler flowmetry in rat; see Fig. 5*A*, *D*, respectively). During hypercapnia, an increased number of astrocyte soma (Fig. 1*D*) displayed increased [Ca^2+^]_i_ with onsets within seconds (Fig. 1*B*,*E*) and variable durations of tens of seconds (Fig. 1*B,F*). While there were no differences between the three groups (astrocyte soma, neuronal soma, and neuropil) with regards to the delay of the hypercapnia-induced Ca^2+^ responses (average Ca^2+^ response delay [Fig. 1*E*]: neuron soma = 12.14 ± 1.19 s (*n* = 33), neuropil = 12.83 ± 4.18 s (*n* = 3), and astrocyte soma = 14.57 ± 1.55 s (*n* = 47)), the average Ca^2+^ response duration (Fig. 1*F*) was found to be significantly longer in astrocytes than in neurons: neuron soma = 119.41 ± 8.82 s (*n* = 33), astrocyte soma = 155.47 ± 8.32 s (*n* = 47) (*p* < 0.05, ANOVA).

### Astrocytic [Ca^2+^]_i_ signals evoke subsequent GSH-dependent PgE_2_ release

Having demonstrated *in vivo* that hypercapnia evokes an increase in astrocyte [Ca^2+^]_i_, we then used a combination of 2PLSM and PgE_2_ measurements using ELISA in acute brain slices to determine the mechanistic links between astrocyte [Ca^2+^]_i_ responses and CBF regulation. Using a biochemical model, we investigated the role of GSH in the generation of PgE_2_.

**Figure 2.**
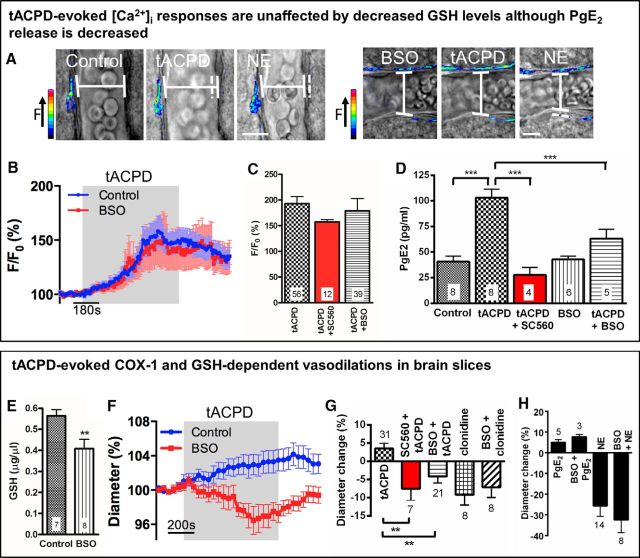
Astrocyte [Ca^2+^]_i_ signals evoke COX-1- and GSH-dependent vasodilations *in vitro*. ***A***, 2PLSM imaging: example Ca^2+^ and arteriole diameter changes in response to tACPD with and without BSO. Images represent overlay of pseudo-colored Ca^2+^ changes and transmitted light images. Dotted line indicates initial vessel diameter. Scale bar, 10 μm. ***B***, Mean time course of increase in astrocyte [Ca^2+^]_i_ in response to tACPD. Colored box represents time of tACPD application. Control, *n* = 56 from 26 rats; BSO, *n* = 39 from 18 rats. ***C***, Mean tACPD-evoked increase in astrocyte [Ca^2+^]_i_. tACPD, *n* = 56 from 26 rats; tACPD + SC560, *n* = 12 from 7 rats; tACPD + BSO, *n* = 39 from 18 rats. ***D***, Mean tACPD-evoked PgE_2_ release, measured by ELISA. Within a group, each experiment (*n*) uses tissue from a different rat (i.e., control, *n* = 8 from 8 rats). ***E***, Mean tissue GSH concentration; data from 4 rats for each group. ***F***, Mean time course of tACPD-evoked change in lumen diameter. Colored box represents time of tACPD application. Control, *n* = 31 slices from 26 rats; BSO, *n* = 21 slices from 18 rats. ***G***, Mean changes in lumen diameter evoked by tACPD and clonidine. tACPD, *n* = 31 slices from 26 rats; SC560 + tACPD, *n* = 7 slices from 7 rats; BSO + tACPD, *n* = 21 slices from 18 rats; clonidine, *n* = 8 slices from 8 rats; BSO + clonidine, *n* = 8 slices from 7 rats. ***H***, Mean changes in lumen diameter evoked by PgE_2_ and NE. PgE_2_, *n* = 5 slices from 4 rats; BSO + PgE_2_, *n* = 3 slices from 3 rats; NE, *n* = 14 slices from 11 rats; BSO + NE, *n* = 8 slices from 7 rats. Data are mean ± SEM. **p < 0.01. ***p < 0.001. *n*, number of experiments conducted or, for calcium measurements, number of astrocyte ROIs analyzed.

Unlike in the *in vivo* situation, it is difficult to reliably evoke astrocyte [Ca^2+^]_i_ signals and vasodilations by applying CO_2_ to acute brain slices. Thus, we needed an alternative method of elevating astrocyte [Ca^2+^]_i_ in acute brain slices. Although the adult mouse (Sun et al., 2013) and rat (Duffy and MacVicar, 1995) have been shown to not express functional mGluR5, bath application of the mGluR agonist tACPD is known to increase astrocyte [Ca^2+^]_i_ in younger animals (Mulligan and MacVicar, 2004). Therefore, tACPD was used to evoke reliable, reproducible astrocyte [Ca^2+^]_i_ elevations in acute brain slices from juvenile rats. To evoke widespread increases in astrocyte [Ca^2+^]_i_, hippocampal-neocortical slices were perfused with tACPD, an mGluR agonist. Application of tACPD (100 μm) to brain slices (from juvenile rats) caused a generalized increase in astrocyte [Ca^2+^]_i_, observed using 2PLSM (Fig. 2*A–C*), that provided us with the ability to measure subsequent synthesis of PgE_2_. Applying tACPD resulted in the formation and efflux of PgE_2_, as measured by ELISA (Fig. 2*D*). The first step in the conversion of AA to PgE_2_ in astrocytes is via COX-1 (Fig. 7) (Takano et al., 2006; Gordon et al., 2008; Font-Nieves et al., 2012). Neurons, in contrast, express COX-2 but not COX-1 (Nogawa et al., 1997). In support of a central role for COX-1, we found that, although the tACPD-evoked increase in astrocyte [Ca^2+^]_i_ was unaltered (Fig. 2*C*) in the presence of the COX-1 inhibitor SC560 (Smith et al., 1998; 100 nm: Blanco et al., 2008), the resulting formation and efflux of PgE_2_, as measured by ELISA, was abolished (*p* < 0.001; Fig. 2*D*). Thus, astrocyte COX-1 activity is required for the subsequent PgE_2_ release in acute brain slices, which is triggered by astrocyte [Ca^2+^]_i_ signals.

**Figure 3.**
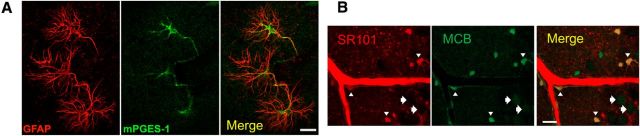
Astrocytes express mPGES-1 and contain high levels of GSH. ***A***, Immunohistochemistry showing astrocytic expression of GSH-dependent mPGES-1 in the CA3 of the hippocampus. Astrocyte marker, GFAP (red), mPGES-1 (green), and merge (yellow). Scale bar, 20 μm. ***B***, MCB-loaded hippocampal-neocortical slices. Astrocytes (identified by SR101, red, white arrowheads) contain higher levels of GSH (as indicated by MCB staining, green) than neurons (white arrows). Merge (yellow). Scale bar, 20 μm.

Downstream of COX-1 the synthesis of PgE_2_ involves the enzyme mPgES-1 (Tachikawa et al., 2012), a form of prostaglandin E synthase expressed in astrocytes (Fig. 3*A*) (Tachikawa et al., 2012) that requires the cofactor GSH (Jakobsson et al., 1999; Murakami et al., 2000). It is known that GSH is present in high levels in astrocytes (Sun et al., 2006; Bragin et al., 2010; Robillard et al., 2011), as detected by staining of brain tissue with MCB, a GSH-sensitive dye (Fig. 3*B*). Therefore, we investigated whether PgE_2_ formation was reduced when GSH levels were depressed. We examined whether there is a reduction in astrocyte [Ca^2+^]_i_-evoked PgE_2_ release in hippocampal slices after treatment with BSO (an inhibitor of γ-glutamylcysteine synthetase) for 2.5 h (Sun et al., 2006), which reduced the tissue GSH concentration by 27% (*p* = 0.009; Fig. 2*E*). When GSH levels were decreased, although there was no change in basal PgE_2_ efflux (Fig. 2*D*) or in the amplitude of tACPD-evoked astrocyte [Ca^2+^]_i_ signals (Fig. 2*A–C*), strikingly the tACPD-evoked PgE_2_ efflux was reduced by 64% (*p* < 0.001; Fig. 2*D*).

### Astrocyte [Ca^2+^]_i_ signals evoke COX-1 and GSH-dependent vasodilations in brain slices

As COX-1 activity (Niwa et al., 2001) and PgE_2_ release (Wagerle and Mishra, 1988; Wagerle and Degiulio, 1994) have been shown to lead to increased CBF in response to hypercapnia, we examined whether COX-1-dependent PgE_2_ release evoked by astrocyte [Ca^2+^]_i_ signals triggered by either tACPD application or IP_3_ uncaging resulted in vasodilations.

Bath perfusion of tACPD induced arteriolar dilation in acute brain slices (Fig. 2*A*,*F*,*G*), which was abolished in the presence of SC560 (*p* < 0.01; Fig. 2*G*), whereas the amplitude of evoked astrocyte [Ca^2+^]_i_ signals was unchanged (*p* > 0.05; Fig. 2*C*). Thus, combined with the results discussed above, these data confirm that astrocyte COX-1 activity and subsequent PgE_2_ release are required for vasodilations in acute brain slices that are triggered by astrocyte [Ca^2+^]_i_ signals.

As previously discussed, downstream of COX-1, the synthesis of PgE_2_ involves the astrocyte-expressed, GSH-dependent, enzyme mPgES-1 (Tachikawa et al., 2012). Therefore, a role for astrocytes in the regulation of arteriole diameter would be supported if [Ca^2+^]_i_-evoked vasodilations were attenuated when GSH levels were depressed. We examined whether there is a reduction in subsequent vasodilations in hippocampal slices after treatment with BSO. When GSH levels were decreased, tACPD-evoked astrocyte [Ca^2+^]_i_ signals were unaltered (Fig. 2*A–C*). However, the vasodilations triggered by these [Ca^2+^]_i_ signals were abolished (Fig. 2*A*,*F*,*G*; *p* < 0.01). Vasoconstrictions evoked by NE (100 μm) or the α_2_ agonist clonidine (10 μm), which act directly on arteriole smooth muscle cells (Busija and Leffler, 1987), were unchanged in the presence of BSO (Fig. 2*A*,*G*,*H*), indicating that arterioles were not damaged by the BSO treatment. Furthermore, BSO treatment did not alter the vasodilation evoked by either 1 μm PgE_2_ (Fig. 2*H*) or high [K^+^] (10 mm), which causes vasodilation by hyperpolarizing arteriole smooth muscle cells (Filosa et al., 2006) (K^+^: 8.6 ± 2.3%, *n* = 5 slices from 5 rats; BSO + K^+^: 6.5 ± 0.8%, *n* = 6 slices from 3 rats, *p* = 0.37).

**Figure 4.**
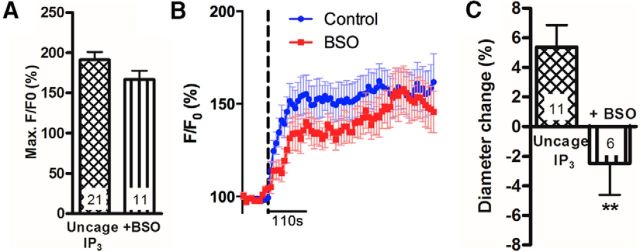
Astrocyte [Ca^2+^]_i_ transient-evoked vasodilations are GSH dependent *in vitro*. ***A***, Mean IP_3_-evoked increases in astrocyte [Ca^2+^]_i_. Control, *n* = 21 from 6 rats; +BSO, *n* = 11 from 4 rats. ***B***, Mean time course of increase in astrocyte [Ca^2+^]_i_. Dotted line indicates time of photolysis of caged IP_3_. *n* as described in ***A***. ***C***, Mean lumen diameter change in response to uncaging of IP_3._ Uncage IP_3_, *n* = 11 slices from 6 rats; +BSO, *n* = 6 slices from 4 rats. Data are mean ± SEM. **p < 0.01. *n*, number of experiments conducted or, for calcium measurements, number of astrocyte ROIs analyzed.

Astrocyte [Ca^2+^]_i_ increases can be triggered by two-photon uncaging of IP_3_ within the cell body of an astrocyte. Using this technique, we directly examined the effect of decreasing GSH levels on astrocyte [Ca^2+^]_i_-evoked arteriole dilations. Astrocytes in hippocampal slices from juvenile rats were bulk-loaded with the caged IP_3_ compound, NV-IP_3_/AM. Two-photon photolysis was used to uncage IP_3_ within an astrocyte soma specifically, generating a [Ca^2+^]_i_ increase within the soma, processes, and endfeet. This local increase in [Ca^2+^]_i_ could evoke an increase in [Ca^2+^]_i_ in nearby astrocytes (Fig. 4*A*,*B* represents local and propagated responses) and elicited vasodilation of the neighboring arteriole (Fig. 4*C*). Although astrocyte [Ca^2+^]_i_ signals were unaltered following BSO treatment to reduce GSH levels (*p* = 0.1; Fig. 4*A*,*B*), dilations were not observed and vasoconstrictions were now evoked (*p* = 0.008; Fig. 4*C*). Thus, when GSH levels are reduced, astrocyte [Ca^2+^]_i_ signals can no longer evoke vasodilations normally triggered by the release of PgE_2_.

### *In vivo* hypercapnia-evoked CBF responses are GSH dependent

**Figure 5.**
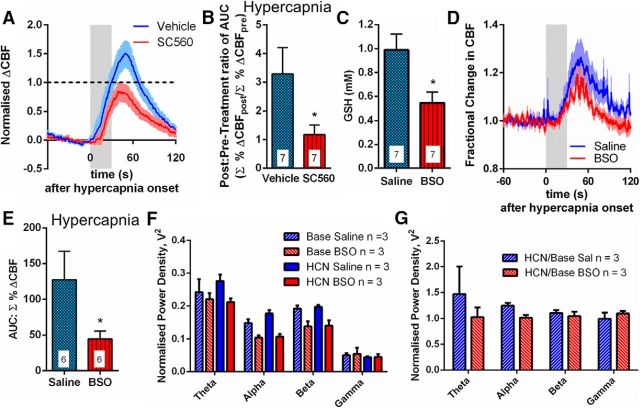
CO_2_ evoked CBF responses *in vivo* are GSH dependent. ***A***, Mean traces of local CBF response to hypercapnia, measured by laser speckle contrast imaging, in vehicle (DMSO)- (blue) and SC560- (red) injected animals. *n* = 7 rats for each group. Colored box represents time of CO_2_ application. Data shown as fractional change with baseline of 0 (baseline taken during 60 s prechallenge) and a pretreatment peak of 1 (black dotted line on graph). ***B***, Mean AUC of CBF response to hypercapnia in the presence of vehicle (DMSO) or SC560 (normalized to pretreatment maxima for each animal). *n* = 7 rats for each group. ***C***, Tissue GSH levels 24 h after injection of BSO or saline into the barrel cortex (*n* = 7 rats). ***D***, Mean trace of local CBF response to hypercapnia, measured by laser Doppler flowmetry, in saline- (blue) and BSO- (red) injected rats. *n* = 6 rats in each group. ***E***, Mean values of AUC of CBF response to hypercapnia. *n* = 6 rats in each group. ***F***, ***G***, Neural activity. Power in frequency bands. ***F***, During baseline (Base) and in response to hypercapnia (HCN) for saline- (blue) and BSO- (red) treated animals. *n* = 3 rats. ***G***, Hypercapnia (HCN)/baseline (Base). Treatment with BSO does not change the effect of hypercapnia on neural activity. *n* = 3 rats. Data are mean ± SEM. * *p* < 0.05.

Having determined in acute brain slices the vasodilatory molecules underlying astrocyte [Ca^2+^]_i_-evoked vasodilations, we examined whether these same enzymes and molecules were involved in the CBF response, which occurs downstream of CO_2_-evoked astrocyte [Ca^2+^]_i_ responses *in vivo*. Hypercapnia *in vivo* evoked a CBF increase in the barrel cortex of adult rat (Fig. 5*A*,*B*,*D*,*E*), whereas neural activity was unchanged (Fig. 5*F*). The calculated area under the curve (AUC) of the CBF response was significantly attenuated by SC560 (*p* = 0.032; Fig. 5*A*,*B*), confirming that COX-1 plays a critical role in hypercapnia-evoked CBF increases *in vivo* (Niwa et al., 2001).

We examined the impact of decreased tissue GSH levels on CO_2_-evoked CBF increases *in vivo*. To lower GSH levels *in vivo*, BSO was injected into rat barrel cortex. After 24 h, tissue GSH levels in the ipsilateral cortex were reduced by 45% (Fig. 5*C*; *p* = 0.018). Treatment with BSO reduced the hypercapnia-evoked CBF response (Fig. 5*D,E*; AUC reduced by 65%, *p* = 0.048). Neural activity was no different in BSO-treated rats compared with saline-treated rats (Fig. 5*G*). Combining all the data described so far suggests that hypercapnia-evoked, astrocyte [Ca^2+^]_i_-related, CBF increases require PgE_2_ release and, thus, are compromised when brain GSH levels are reduced.

**Figure 6.**
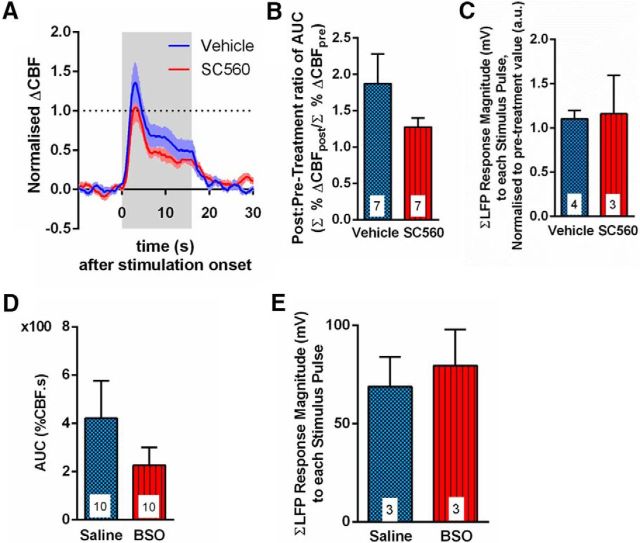
CBF responses to whisker pad stimulation *in vivo* are independent of GSH. ***A***, Mean time course of local CBF response to whisker pad stimulation, measured by laser speckle contrast imaging, in vehicle (DMSO)- (blue) and SC560- (red) injected rats. Colored box represents time of stimulation. Dotted black line indicates pretreatment peak of 1. ***B***, Mean AUC of the CBF response to whisker pad stimulation. *n* = 7 rats for each group. ***C***, Mean neural response (LFP) magnitude to whisker pad stimulus (summed over total 16 s length of stimulus). Responses are normalized to the first pulse response for each rat. *n* = 4 DMSO-treated rats; *n* = 3 SC560-treated rats. ***D***, Mean AUC of the whisker pad stimulation-evoked CBF response in saline- (blue) and BSO- (red) injected rats. *n* = 10 rats for each group. ***E***, Mean neural response (LFP) magnitude to whisker pad stimulation (summed over total 16 s length of stimulus). Responses are normalized to the first pulse response for each rat. *n* = 3 rats in each group. Data are mean ± SEM.

This finding was specific to hypercapnia-evoked CBF increases. We examined the impact of decreased tissue GSH levels *in vivo* on functional hyperemia in the somatosensory cortex. Whisker pad stimulation (10 Hz) evoked a blood flow increase in the barrel cortex (Fig. 6*A*). In agreement with previous findings (Niwa et al., 2000), inhibiting COX-1 with SC560 had no effect on either the CBF response to whisker pad stimulation (Fig. 6*A*,*B*; *p* = 0.10) or evoked neural activity (LFP) (Fig. 6*C*; *p* = 0.91). Furthermore, the AUC of the stimulation-evoked CBF response was not significantly different in BSO-treated animals (Fig. 6*D*; *p* = 0.14) compared with saline-treated animals, demonstrating that the CBF response is not GSH-sensitive. The magnitude of the neural response to whisker pad stimulation was unaffected by BSO (Fig. 6*E*; *p* = 0.68). These results indicate that, under these experimental conditions, COX-1 and GSH play little, if any, role in the CBF response to somatosensory stimulation. These findings confirm that several different pathways exist that account for CBF regulation under differing conditions and in response to different stimuli.

## Discussion

**Figure 7.**
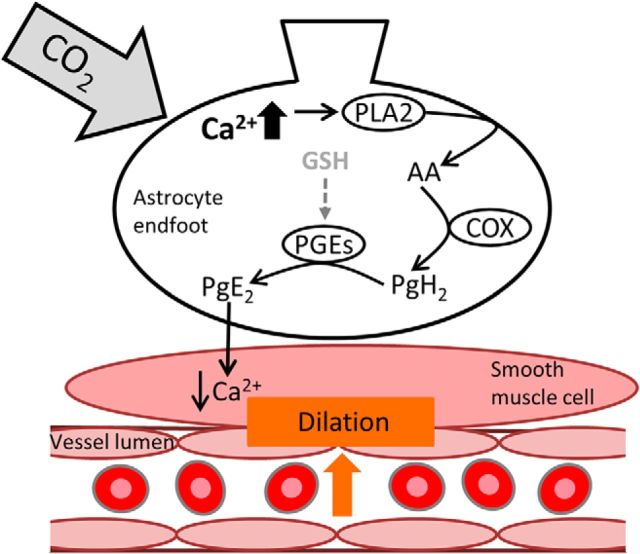
Increases in astrocytic [Ca^2+^]_i_ may lead to GSH-dependent, PgE_2_-mediated vasodilation. Schematic diagram depicting how CO_2_-evoked increases in astrocytic [Ca^2+^]_i_ may lead to PgE_2_-mediated vasodilation. As a result of elevated [Ca^2+^]_i_, PLA_2_ is activated. PLA_2_ generates AA from the plasma membrane. AA can be processed locally by COX enzymes to produce AA derivatives, such as prostaglandin H_2_ (PgH_2_). PgE_2_ is produced from PgH_2_ by the enzyme PGEs, which requires GSH as a cofactor (Jakobsson et al., 1999; Murakami et al., 2000; Tanioka et al., 2000). PgE_2_ is released from astrocyte endfeet, which are apposed to the smooth muscle layer surrounding arterioles, resulting in activation of K^+^ channels, a decrease in Ca^2+^ entry into the smooth muscle cell and vasodilation.

We demonstrate a novel mechanism of CBF regulation involving astrocytes, which is GSH dependent. Previously, Niwa et al. (2001) demonstrated that hypercapnia-evoked CBF increases are principally COX-1 dependent. In this study, we examined the mechanism of such CBF regulation, both upstream and downstream of hypercapnia-evoked increases in COX-1 activity (Fig. 7). We demonstrate *in vivo* that, upstream of evoked COX-1 activity, CO_2_ increases [Ca^2+^]_i_ in astrocytes. These data demonstrate a new signal (hypercapnia) that activates astrocyte calcium and specifically identify the involvement of astrocytes in the regulation of CBF in response to changes in arterial CO_2_.

*In vitro*, using brain slices from juvenile animals in which it is possible to examine calcium signals by bulk loading a calcium indicator dye, we confirm that increased astrocyte [Ca^2+^]_i_ results in the subsequent release of PgE_2_ and vasodilation which are COX-1 activity-dependent (Fig. 7). Our assumption that the evoked response in juvenile rat slices is the same as in adult rat with respect to COX-1 dependence is supported by the fact that the same COX-1 dependence has been shown in adult mice (Takano et al., 2006). We demonstrate that these findings hold *in vivo*, confirming previous findings in adult mice (Niwa et al., 2001). Astrocytic endfeet, which are apposed to cerebral vascular smooth muscle, express all the machinery necessary for PgE_2_ synthesis (COX-1) (Takano et al., 2006; Gordon et al., 2008), mPgES-1 (Fig. 3*A*) (Tachikawa et al., 2012), and GSH: (Fig. 3*B*) (Sun et al., 2006; Bragin et al., 2010; Robillard et al., 2011), providing further evidence for the involvement of astrocytes in the regulation of CBF responses to hypercapnia. mPgES, an enzyme selectively expressed in astrocytes compared with neurons (Tachikawa et al., 2012), is the enzyme responsible for producing PgE_2_ downstream of COX-1 activity. Intriguingly, the formation of PgE_2_ is regulated by the availability of GSH in astrocytes, as PgES requires GSH as a cofactor (Jakobsson et al., 1999; Murakami et al., 2000). *In vitro*, we demonstrate that astrocyte [Ca^2+^]_i_-evoked vasodilations are attenuated when GSH levels are depleted, whereas *in vivo*, we demonstrate that CO_2_-evoked CBF increases occur via a GSH-dependent mechanism. As astrocytes contain high levels of GSH (Fig. 3*B*) (Sun et al., 2006; Bragin et al., 2010; Robillard et al., 2011), the dependence of the CO_2_-evoked CBF response on GSH is further evidence of astrocytic involvement. Together, our findings suggest a novel mechanism of astrocyte-evoked CBF regulation, which is GSH dependent. We propose that increased CO_2_ levels evoke [Ca^2+^]_i_ responses in astrocytes, subsequently activating a signaling pathway, involving COX-1 and the GSH-dependent PgES, which results in the release of the vasodilator PgE_2_. Thus, an increase in CO_2_ results in an astrocyte-driven, GSH-dependent vasodilation (Fig. 7).

This GSH-dependent mechanism of CBF regulation exists alongside other COX-1 and GSH-insensitive mechanisms. For example, we found no effect of blocking COX-1 activity or of lowering GSH levels on CBF responses following 10 Hz whisker pad stimulation. Although it is possible that an astrocyte calcium response (and, thus, a GSH-sensitive mechanism of CBF regulation) may be evoked by an intense sensory stimulus (Schulz et al., 2012; Sekiguchi et al., 2016), our results are in agreement with previous work suggesting that COX-1 is involved in CBF responses to hypercapnia (Niwa et al., 2001) but not sensory stimulation (Niwa et al., 2000). Although we saw no evidence that this pathway was important for functional (neuronal activity-evoked) increases in CBF under our experimental conditions, astrocytes appear to be an important intermediary for physiological (hypercapnia-evoked) increases in CBF. Our findings suggest that CBF regulation may involve astrocytes, and their [Ca^2+^]_i_ signals, under certain conditions and not under others.

Previous studies have provided evidence for several mechanisms linking astrocyte [Ca^2+^]_i,_ increases and changes in CO_2_ concentration. For example, within the respiratory center, increased astrocyte [Ca^2+^]_i,_ and astrocytic release of ATP can be triggered by CO_2_-evoked decreases in pH (Gourine et al., 2010). This [Ca^2+^]_i,_ increase may be the result of increased Na^+^/HCO_3_^−^ cotransport and reversal of Na^+^/Ca^2+^ transport (Turovsky et al., 2016). It is unknown whether this mechanism also occurs within the cortex. Alternatively, increased CO_2_ can evoke hemichannel-mediated release of ATP (Huckstepp et al., 2010), which may act on astrocytic purinergic receptors to elicit an increase in [Ca^2+^]_i_ (Pelligrino et al., 2011). Depending on the mechanism linking increases in CO_2_ to astrocyte [Ca^2+^]_i_ responses, therefore, astrocytes could act as either a pH or CO_2_ sensor. Although it is beyond the scope of this paper to determine the link between an increase in CO_2_ and the increase in astrocyte [Ca^2+^]_i_, we have demonstrated that the depletion of GSH levels leads to a reduction in the ability of astrocytes to release PgE_2_ following such a rise in [Ca^2+^]_i,_ and so reduces their ability to evoke vasodilation in response to hypercapnia. This occurs because astrocytes express GSH-dependent mPgES-1.

Our finding that CBF responses to increased CO_2_ are GSH-sensitive suggests that global CBF regulation, which is sensitive to the partial pressure of arterial CO_2_ (Ainslie and Duffin, 2009), will be affected in conditions where GSH levels are depleted. Alterations in the redox status of brain tissue that are ultimately linked to cellular GSH levels have been observed in numerous neurological and psychiatric disorders (Slivka and Cohen, 1993; Tohgi et al., 1995, 1999; Ansari and Scheff, 2010; Zhang et al., 2012; Kulak et al., 2013). Therefore, the impact of changes in GSH levels on the sensitivity of astrocyte regulation of vasodilation could contribute to several CNS pathologies. Thus, it is critical to understand the signaling pathways underlying changes in CBF, both in health and disease.

It has previously been shown that, in addition to astrocytic production of PgE_2_ via COX-1/mPgES activity, neurons (which express COX-2 but not COX-1) (Nogawa et al., 1997; Lecrux et al., 2011), are capable of producing COX-2-derived PgE_2_ (which contributes to neurovascular coupling) (Lecrux et al., 2011; Lacroix et al., 2015). In this study, we used a pharmacological approach to increase astrocyte [Ca^2+^]_i_ and to inhibit either the *de novo* synthesis of glutathione or the activity of COX-1, specifically, to demonstrate that, downstream of an increase in astrocyte [Ca^2+^]_i_, COX-1 activity and glutathione are required for vasodilation to occur. However, as this pharmacological approach lacks cellular specificity, a contribution of neuronally produced PgE_2_ to the hypercapnia-evoked CBF response cannot be completely excluded. Nevertheless, our conclusion that astrocyte COX-1-derived PgE_2_, rather than neuronal COX-2-derived PgE_2_, is involved in the CBF response to hypercapnia is in agreement with previous findings (Niwa et al., 2001). Future studies could use an astrocyte-specific genetic strategy (such as cell-specific knock-out) (Casper et al., 2007) to confirm that hypercapnia-evoked vasodilations, occurring downstream of astrocyte [Ca^2+^]_i_ responses, are dependent on astrocyte glutathione levels and COX-1 activity.

In conclusion, we demonstrate a novel mechanism by which astrocytes detect hypercapnia and, via [Ca^2+^]_i_ signals, increase CBF in response to CO_2_. Astrocytes are therefore poised to detect the metabolic activity of neurons and to modify vascular tone appropriately to deliver glucose and O_2_. This important pathway may be impaired in conditions in which oxidative stress reduces GSH levels in astrocytes, leading to impaired CBF responses and altered vascular readouts of neural activity.
